# Evaluation of the effect of nano‐graphene oxide on shear bond strength of conventional and resin‐modified glass ionomer cement

**DOI:** 10.1002/cre2.789

**Published:** 2023-09-22

**Authors:** Parisa Ghodrati, Farahnaz Sharafeddin

**Affiliations:** ^1^ Department of Operative Dentistry, Biomaterials Research Center, School of Dentistry Shiraz University of Medical Sciences Shiraz Iran; ^2^ Department of Operative Dentistry, School of Dentistry Shiraz University of Medical Sciences Shiraz Iran

**Keywords:** bond strength, glass ionomer, graphene oxide, resin modified glass ionomer

## Abstract

**Objectives:**

Recently, nano‐graphene oxide (nGO), a material with unique mechanical properties, has been introduced to improve the properties of glass ionomer cement (GIC). The purpose of this study was to investigate the effect of adding nGO on the shear bond strength (SBS) of conventional (CGIC) and resin‐modified GIC (RMGIC).

**Methods:**

Sixty intact molars were mounted and their occlusal surface was cut at a depth of 1 mm below the dentinoenamel junction. 1 wt.% and 2 wt.% of nGO (US Research Nanomaterials, Inc.) were added to CGIC and RMGIC (GC Corporation). The samples were randomly divided into six groups (*n* = 10), including 1: CGIC,  2: CGIC + 1% GO, 3: CGIC + 2% GO, 4: RMGIC, 5: RMGIC + 1% GO, and 6: RMGIC + 2% GO. Plastic molds were placed on the surface of the dentin pretreated with 10% polyacrylic acid (GC Corporation) and filled with prepared cement according to the manufacturer's instruction. After 24 h of storage in an incubator, the SBS test was done by the universal testing machine. Data were analyzed using two‐way analysis of variance and post hoc Tukey tests (*p* < .05).

**Results:**

In the group of CGIC, mean SBS was significantly lower than all other study groups (*p* < .001), and groups 5 (RMGIC + 1% GO) and 6 (RMGIC + 2% GO) showed significantly higher values compared to all other study groups (*p* < .001). However, the difference between groups 2 and 3, as well as the difference between groups 5 and 6, was not significant (*p* = .999 and*p* = .994, respectively). RMGI groups had significantly higher SBS than their corresponding CGIC groups.

**Conclusions:**

The addition of 1% and 2% nGO significantly increased the SBS of CGIC and RMGIC to the dentin, which can be considered as a promising point for wider clinical application of this material.

## 1 INTRODUCTION

Glass ionomer cement (GIC) was presented as a restorative material with various clinical applications such as restoration of milk teeth, Class 3 and Class 5 cavities, and non‐carious lesions (Vasei & Sharafeddin, 2021). What distinguishes GIC from other restorative materials is its unique chemistry, which allows bonding to the enamel and dentin and protects against tooth decay in the margins of restoration by releasing fluoride (Sun et al., 2018). GIC also has a low coefficient of thermal expansion and acceptable esthetics and is relatively easy to use (Amin et al., 2021). However, its poor mechanical properties, such as low fracture strength, low resistance to wear, and low toughness, limit its wide application in dentistry as a restorative material in stress‐bearing areas. Another limitation of GIC is sensitivity to moisture and drying during the initial setting reaction (Sharafeddin & Bahrani, 2020). To improve the clinical application of this cement, resin was added to its formula, which improved the physical and mechanical properties of CGIC. The resin‐modified GIC (RMGIC) is polymerized through light curing and, therefore, has the advantages of increasing the working time, controlling the setting process, and improving esthetics (Sharafeddin et al., 2021).

Different materials such as fibers, nanoparticles, and zirconia have been used to strengthen the mechanical properties of GICs. Recently, fillers with nanometer size have been added to improve the properties of GICs (Sharafeddin et al., 2019). Nanotechnology means the creation of materials with dimensions less than 100 nm (Ge et al., 2018).

Recently, researchers have focused on nanomaterials from the graphene family because of their great mechanical and biological properties (AlFawaz et al., 2020; Ge et al., 2018). Graphene has a two‐dimensional single‐layer compact structure consisting of hybridized sp2‐carbon atoms with a hexagonal arrangement similar to a honeycomb. Graphene‐based materials have a high surface area and are chemically and thermally stable. Among the materials derived from graphene, graphene oxide (GO) is of particular importance, which is also synthesized through the oxidation of graphite. Generally, graphene is known as a hydrophobic material, but GO is hydrophilic due to the presence of oxygen in the chemical structure of its functional groups (Liu et al., 2022).

In the studies conducted up to now, researchers have used GO in dentistry due to its unique properties in the fields of antimicrobial, new cancer treatment techniques, bone and tissue regenerative treatments, as a drug carrier, and for improving the physical and mechanical properties of dental biomaterials (Bonilla‐Represa et al., 2020).

In a study, it was shown that adding fluorinated graphene (FG) to CGIC increased its hardness and resistance to abrasive wear (Liu et al., 2021). In another report, it was inferred that the addition of GO to RMGIC improved its shear bond strength (SBS) (Al‐Qahtani, 2021). In the studies done so far, the effect of different percentages of GO (0.5%, 1%, 2%, 4%) (Sun et al., 2018) on the mechanical properties of different materials such as GICs and adhesives have been investigated. However, to the best of our knowledge, there have been very few studies on the effect of GO on the SBS of GICs.

Therefore, we aimed to investigate the effect of 1 wt.% and 2 wt.% of nano‐graphene oxide (nGO) on the SBS of CGIC and RMGIC. The null hypothesis of this article was the addition of nGO to CGIC and RMGIC would not improve its SBS to the dentin.

## 2 MATERIALS AND METHODS

### 2.1 Preparation of dental samples

In this experimental study with the ethics code of IR.SUMS.DENTAL.REC.1401.029, 60 human molars that had no caries, restorations, or fractures and were extracted for orthodontic reasons with the informed consent of the patients were used. After extraction, the teeth were washed with water and cleaned using an ultrasonic scaler. After being disinfected with the distilled water solution containing 0.1% thymol, the teeth were kept in the distilled water at a temperature of 4°C until the required time (Sharafeddin, Alavi, et al., 2020). The teeth were mounted in self‐hardening acrylic resin (Acropars) in a metal mold (length:30 × width:20 × height:15 mm) up to 2 mm below the cementoenamel junction, so their occlusal surfaces were parallel to the acrylic resin surface and were available for surface preparation. Next, the acrylic resin was polymerized and removed from the metal molds; then, the occlusal surface of the teeth was horizontally sectioned at a depth of 1 mm below the dentinoenamel junction (DEJ) by a diamond disc (D and Z) and high‐speed handpiece under water cooling to expose the smooth surface of the dentin, which was further polished with standard 600 grit silicon carbide sandpaper. Then, the samples were randomly divided into six groups (*n* = 10): group 1 (CGIC), group 2 (CGIC + 1%GO), group 3 (CGIC + 2% GO), group 4 (RMGIC), group 5 (RMGIC + 1% GO), and group 6 (RMGIC + 2% GO). The surface of all samples was conditioned with 10% polyacrylic acid (Dentin Conditioner; GC Corporation) for 20 s and then gently washed with water and dried with a cotton pallet.

### 2.2 Addition of nGO to GICs

Powder preparation was done using a digital scale (A & D; GR+360) with an accuracy of ±0.0001 g to weigh the powder. The desired amounts of nGO powder (US Research Nanomaterials, Inc.) were added to each CGI and RMGI powder (GC Corporation) to prepare the mixtures of (CGI/RMGI + 1 wt.% nGO) and (CGI/RMGI + 2 wt.% nGO) (Chen et al., 2020). To make a uniform and homogenous mixture of the prepared powder, they were poured into empty and clean amalgam capsules and vibrated for 20 s in the amalgamator (Ultramat 2; SDI). Thereupon, to ensure uniform mixing, two trained technicians examined the prepared powder under a stereomicroscope (BestScope; BS‐3060C) with x40 magnification; since nGO is dark and GI is white, the distribution of nGO can be somewhat distinguished. In the case of nonuniform distribution of nGO particles and observation of lump‐like and accumulated particles of nGO, which was confirmed by both technicians, the powder was removed, and a new mixture was prepared and checked.

### 2.3 Preparation of GIC samples

According to the manufacturer's instructions, one scoop of powder was mixed with a drop of liquid for groups of CGIC, and one scoop of powder was mixed with two drops of liquid for the RMGIC groups on a cold glass slab by a plastic spatula for 25 s. To obtain the same shape and size of all the samples, the plastic cylindrical molds with a diameter of 3 mm and a height of 2 mm were inserted on the treated surface of the dentin and filled with the prepared cement by a thin composite instrument (Sharafeddin, Alavi, et al., 2020). The same researcher did the cement mixing and filling for all the samples. After that, in the CGIC groups, it took 5.5 min to set completely, and the RMGIC groups were cured for 20 s by the LED light curing device (BlueLEX; Monitex) with a light intensity of 1200 mW/cm^2^ according to the manufacturer's instructions at the occlusal direction and with a distance of 1 mm from the surface of the cement. In all groups, the cement was covered with a transparent matrix during setting. The plastic molds and matrix were carefully removed after the specimens were set, and then a layer of varnish (GC Corporation) was applied to the restoration surfaces to protect against moisture, according to the manufacturer's instructions (Sharafeddin, Alavi, et al., 2020).

### 2.4 SBS test

**Figure 1:**
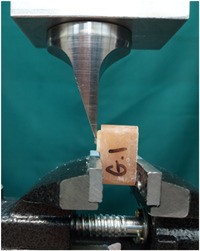
The sample under the application of force for the SBS test in the universal testing machine. SBS, shear bond strength.

All the samples were kept in an incubator (Nuve) with a temperature of 37°C and a humidity of about 100% for 24 h and then subjected to thermocycling (PC300; Vafaei) for 500 cycles at 5°C and 55°C with a dwell time of 30 s and transfer time of 30 s between the baths. Then, a universal testing machine (Zwick/Roell; Z020) was utilized for SBS analysis. A crosshead speed of 1 mm/min was the maximum force applied to the bonded specimens until the failure of the bond happened (Figure 1). The maximum load to failure was recorded (N) and the SBS was measured in MPa.

### 2.5 Analysis of the failure mode

The debonded samples were observed under a stereomicroscope (BestScope; BS‐3060C) with ×40 magnification to determine the type of failure. The failure modes, according to similar articles, were as follows: (1) cohesive failure in the dentin, (2) cohesive failure in the cement, (3) adhesive failure at the interface (bonded surface between dentin and cement), and (4) mixed failure (failure in the bonded surface that extends into the dentin and/or cement) (Talip et al., 2017).

All the information about materials used in this study is shown in Table 1.

**Table 1:** Composition of the materials utilized in this study.

Materials	Composition	Manufacturer's
Conventional glass ionomer cement, Fuji II (CGIC)	Powder: fluoroaluminosilicate glass Liquid: polyacrylic acid, itaconic acid, tartaric acid, maleic acid, and water	GC Corporation Tokyo, Japan
Resin‐modified glass ionomer cement, Fuji II LC (RMGIC)	Powder: fluoroaluminosilicate glass Liquid: liquid: polyacrylic acid: 2‐hydroxyl ethyl methacrylate, urethane dimethacrylate, camphorquinone, and distilled water	GC Corporation Tokyo, Japan
Dentin conditioner	10% polyacrylic acid	GC Corporation Tokyo, Japan
Nano‐graphene oxide powder	Graphene oxide nano‐platelets (99+%, 3.4−7 nm, 6−10 layers)	US Research Nanomaterials, Inc., Houston, USA
GC Fuji Varnish	Isopropyl acetate 50%−70% Acetone 20%−30%	GC Corporation Tokyo, Japan

### 2.6 Statistical analysis

All data recorded in the present study were statistically analyzed using SPSS (IBM statistics version 26). The values of SBS were described in mean ± standard deviation (SD) and compared using two‐way analysis of variance (ANOVA). Tukey's post hoc test was done for multiple comparisons. Failure modes between study groups were compared using Fisher's exact test. The mean difference was considered significant at *p* < .05.

## 3 RESULTS

**Figure 2:**
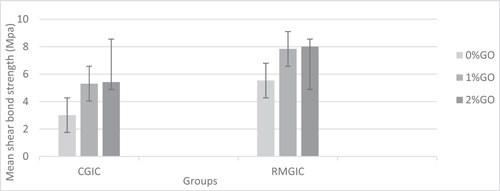
Mean shear bond strength (Mpa). CGIC, conventional glass ionomer cement; GO, graphene oxide; RMGIC, resin‐modified glass ionomer cement.

The means and SD of SBS in all groups (*n* = 10) are presented in Table 2 and Figure 2. According to the two‐way ANOVA for SBS, there was a significant difference between all research groups (*p* < .001), as shown in Table 2.

**Table 2:** Means and standard deviations for shear bond strength (Mpa).

Study groups (*n* = 10)	SBS (Mpa) (mean ± SD)	*p* Value*
1 (CGIC)	3.01 ± 0.79^a^	<.001
2 (CGIC + 1% GO)	5.30 ± 0.48^b^	
3 (CGIC + 2% GO)	5.42 ± 0.60^b^	
4 (RMGIC)	5.53 ± 0.55^b^	
5 (RMGIC + 1% GO)	7.84 ± 0.83^c^	
6 (RMGIC + 2% GO)	8.01 ± 0.97^c^	

*Note*: The mean values of the different letters were statistically significant (post hoc Tukey's test).

Abbreviations: ANOVA, analysis of variance; CGIC, conventional glass ionomer cement; GO, graphene oxide; RMGIC, resin‐modified glass ionomer cement; SBS, shear bond strength.

* Two‐way ANOVA.

Multiple comparisons using post hoc Tukey's test showed that SBS in group 1 (CGIC) was significantly lower than that in all other study groups (*p* < .001), and the results of groups 2 (CGIC + 1% GO) and 3 (CGIC + 2% GO) were not significantly different (*p* = .999). Moreover, the mean bond strength obtained in groups 5 (RMGIC + 1% GO) and 6 (RMGIC + 2% GO) did not differ significantly from each other (*p* = .994) but showed significantly higher values than all other study groups (*p* < .001). In addition, compared to group 1, group 4 demonstrated a statistically higher bond strength (*p* < .001), but the difference between groups 4 and 2, as well as that between groups 4 and 3, was not significant (*p* = .981 and *p* = .999, respectively). RMGI groups had significantly higher SBS than their corresponding CGIC groups (Table 2).

**Figure 3:**
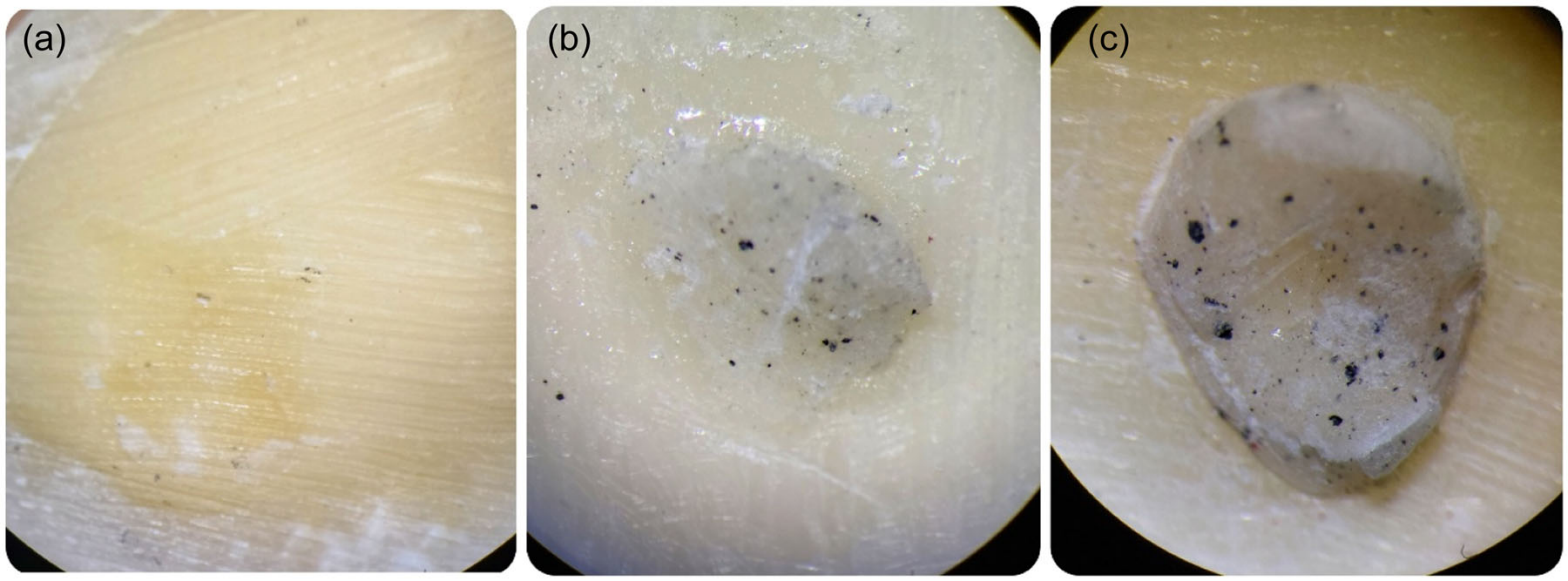
Failure modes of SBS. Adhesive (a), mixed (b), cohesive in cement (c). SBS, shear bond strength.

Table 3 shows the percentage of the distribution of failure mode in the subjects in each group. In group 1, the type of failure was only adhesive, while in all other experimental groups, both adhesive and mixed failure modes were seen. However, cohesive failure in cement was observed only in groups 5 and 6 (Figure 3). According to Fisher's exact test, there was no significant difference between the groups (*p* = .373).

**Table 3:** Percentage of the distribution of failure mode in all research groups.

Failure mode (%)	Group 1 (CGIC)	Group 2 (CGIC + 1% GO)	Group 3 (CGIC + 2% GO)	Group 4 (RMGIC)	Group 5 (RMGI + 1% GO)	Group 6 (RMGI + 2% GO)	*p* Value^a^
Cohesive in dentin	0	0	0	0	0	0	
Cohesive in cement	0	0	0	0	10	10	.373
Adhesive	100	70	60	90	70	60	
Mixed	0	30	40	10	20	30	
Total	100	100	100	100	100	100	

Abbreviations: CGIC, conventional glass ionomer cement; GO, graphene oxide; RMGIC, resin‐modified glass ionomer cement.

^a^ Fisher's exact test.

## 4 DISCUSSION

One of the important features of restorative materials is their ability to bind to the tooth tissue. GIC, which is introduced as an adhesive restorative material, can create adhesion between the carboxyl groups of polyacrylic acid and hydroxyapatite on the surface of the tooth (Sharafeddin, 2017). The requirement of the restorative material that can be used in the posterior permanent teeth is to bear the chewing force of at least 125 MPa (Sun et al., 2018). Recent studies indicate that adding nanoparticles to GIC improves its mechanical properties (Moheet et al., 2019). GO is made by processing graphene with oxygen‐containing groups such as hydroxyl (−OH) and carboxyl (−COOH) (Qi et al., 2021).

The findings of this research showed that both 1 wt.% and 2 wt.% of nGO significantly increased the SBS of CGIC and RMGIC, which is in line with the results of previous studies (Alnatheer et al., 2021; Bin‐Shuwaish et al., 2020). Thus, the null hypothesis is rejected.

Al‐Fawaz et al. (2020) in their study concluded that adding 0.5 wt.% and 2 wt.% of nGO increased the micro tensile bond strength (TBS) of the adhesive containing 5 wt.% nano‐hydroxyapatite (nHA) and also improved the stability and durability of the adhesive after thermocycling. The bond strength values obtained by them are higher compared to those in our study, which can be due to various reasons, including the presence of nHA. HA increases the surface area to create adhesion with the tooth and also strengthens the mechanical properties of the adhesive (Melo et al., 2013). Another important point is the difference between adhesive and GICs in the type of adhesion to the dentin. Commercial adhesives create a stronger bond with the dentin than GICs by creating a stronger hybrid layer and also due to lower viscosity and greater penetration into the interspaces of the demineralized dentin (Fröhlich et al., 2020). Nevertheless, we used 10% polyacrylic acid to condition the dentin, which causes the partial removal of the smear layer and the demineralization of the upper surface dentin, thereby enabling the establishment of micromechanical adhesion and the formation of a shallow hybrid layer, which leads to the improvement of the bond strength (Nicholson, 2016). It is also worth noting that we used the SBS test because chewing forces around the mouth are a combination of compressive, tensile, and shear that are applied to the restorative material and tooth tissue, but most of them include shear forces (Salem & Asaad, 2019). It can be predicted that if micro‐tests are used instead of macro, much higher bond strength values are obtained (Ismail et al., 2021). Although it can be assumed that just as GO improves the bond strength of dental adhesives, it can also be effective in increasing the bond strength of GICs.

In the current study, increasing the content of nGO from 1 wt.% to 2 wt.% increased the SBS, which is in the same line with some previous research. Bin‐Shuwaish et al. (2020) in their study evaluated the effect of 0.5 wt.% and 2 wt.% of silanized nano‐graphene oxide (SGO) on the TBS of adhesive and found that the increase in SGO content improved the bond strength of the adhesive. Through SEM (scanning electron microscope) analysis, they observed that the increase of the amount of SGO filler in the adhesive clearly could be traced in the hybrid layer and in the dentin tubules, and the GO particles in the resin matrix buried in the dentin and in the hybrid layer had many interactions. They attributed this result to the hydrophilic nature of the GO, which leads to more flow and deeper penetration into the dentin, thereby providing more mechanical support to the hybrid layer (Bin‐Shuwaish et al., 2020). In another study, SEM analysis of modified dentin‐adhesive with GO at the interface revealed the uniform dispersion of graphene particles and its deep penetration in the hybrid layer. Moreover, the analysis of EDX (energy‐dispersive X‐ray spectroscopy) revealed the carbon and oxygen peaks that indicated the presence of GO in the resin tags. They pointed out that the two‐dimensional structure of the GO sheet with a large surface area was able to positively interact with other fillers, which improved its mechanical strength and bond strength. It was also shown that the hydrophilic nature of GO, as an adhesive filler, can cause resistance to moisture and improve its bond strength (Alshahrani et al., 2020). Since the presence of sufficient moisture during the GICs setting and maturation plays an important role in this process (Sidhu & Nicholson, 2016), in our study, it is also possible that the hydrophilic properties of GO, as well as the optimal interactions with dentin resulting from its structure, improve the micromechanical/chemical adhesion of self‐adhesive GICs to the dentin.

In the study conducted by Alnatheer et al. (2021), the effect of adding 0.25 wt.% and 0.5 wt.% of silanized nGO to the adhesive for bonding orthodontic brackets was examined. Contrary to our study, they found that 0.5 wt.% of GO significantly reduced the SBS compared to 0.25 wt.% and attributed this reduction to the negative effect of GO on the degree of conversion (DC) of the adhesive. Adding nano‐fillers to light polymerizable materials can act as a barrier to the deep penetration of the curing light because these nano‐fillers, especially when they are the same size as the wavelength of the curing light, scatter it and limit the DC. Therefore, the amount and size of nano‐fillers affect the rheological characteristics of polymeric materials; for example, increasing the amount of nano‐fillers due to the increase in specific surface area will make the cement more viscous, which in turn can have a negative effect on the bond strength (Rezvani et al., 2019). Although the DC of RMGIC containing nGO was not investigated in our study, adding 1 wt.% and 2 wt.% of nGO to RMGIC did not decrease the bond strength, which could be due to the acid−base reaction and the establishment of a chemical bond with hydroxyapatite of the dentin in addition to free radical polymerization of resin monomers in RMGIC and its micromechanical bond with the dentin.

A study has pointed out that nano‐sized fillers can be effective in lower concentrations due to their high specific surface area (Li et al., 2013). However, recent studies reported that increasing the amount of nano‐fillers up to 2% did not significantly reduce the DC (Alnatheer et al., 2021). A key point to improve the efficiency of adding the fillers to the cement is the uniform distribution of the fillers in the cement; otherwise, the nonuniform distribution will lead to the creation of cracks and porosity in the cement (Mortazavi et al., 2012). In a study, Sun et al. (2018) assessed the effects of different contents of FG (0.5 wt.%, 1 wt.%, 2 wt.%, and 4 wt.%) on the flexural strength, compressive strength, and Vickers microhardness of GIC; they concluded that the mechanical properties of GIC improved by increasing the FG content, except in the group which contained 4%, which showed a decrease compared to the group containing 2%. They stated that adding too much filler would cause their agglomeration and consequently their nonuniform distribution, which in turn leads to the poor bonding of the filler to the cement matrix and the formation of voids and micro‐cracks. In our study, uniform distribution of the nGO up to 2 wt.% was done by hand mixing and vibrating in an amalgamator; then, the mixed powder was examined using a stereomicroscope. However, while mixing the powder, which contained nGO with the liquid, the accumulated particles of nGO were observed. These agglomerated masses might have increased with the addition of 2 wt.% nGO compared to 1 wt.%. In the present study, adding 2% of nGO showed values of bond strength comparable to 1% of that. It should be noted that by adding small amounts of nano‐filler to GIC, there are still enough binding sites for polyacrylic acid to create polysalt bridges and cross‐links, which leads to cement matrix strengthening (Chen et al., 2020).

In some published studies, the hypothesis of crack bridging has been proposed to justify the effect of GO on the mechanical properties of GIC, which includes four different aspects (Gao et al., 2014). Crack bridging refers to the bridging of graphene to the surface or the crack on the opposite side, thereby reducing the crack propagation force. When the shear force is greater than the strength of the interface, the friction between the graphene and the matrix disrupts their relative movement, and the cement matrix needs more energy to pull out the nano‐filler. This phenomenon expresses the pullout mechanism. The two‐dimensional structure of nano‐graphene sheets causes the force to be transferred from one graphene to another, and by creating a complex path for crack propagation, it causes crack deflection. The crack tip protection mechanism occurs when a crack propagates to graphene; also, because the energy is insufficient to create a crack at the interface, the crack is limited to propagate and stops (Sun et al., 2018). In the case of nGO filler, it can be concluded that by improving the mechanical properties of GIC, the bond strength can also be improved.

According to our results, the SBS of RMGIC was significantly higher than that of CGIC, which is in the same line with some previous reports (El Wakeel et al., 2015; Sibal, 2016; Techa‐Ungkul & Sakoolnamarka, 2021). However, there was no significant difference between RMGIC and CGIC + 1% GO, nor between RMGIC and CGIC + 2 wt.% GO, which can be due to the increase in the bond strength of the CGIC as a result of the addition of nGO; nevertheless, the bond strength of the RMGI containing 1 wt.% and 2 wt.% of nGO was significantly higher than their corresponding CGIC groups. One of the reasons for the greater bond strength of RMGIC compared to CGIC is its speed of adsorption on the dentin and its polymerization reaction, which has a higher rate of adsorption on the surface of the dentin due to the light curing process and increased ion exchange. Also, due to the free radical polymerization, it has a secondary bond between polymers (Nicholson, 2016). Another reason is the presence of a hydrophilic monomer hydroxyethyl methacrylate in the RMGI, which leads to improved wettability and toughness and also causes better micromechanical and chemical bonding to the dentin (Techa‐Ungkul & Sakoolnamarka, 2021).

In this study, the most common type of bond failure in all groups was adhesive, which confirms the results of some previous studies (Abdulkader & Aljubori, 2022). In CGIC and RMGIC groups, by the addition of nGO, mixed and cohesive failure in the cement was increased, which can be due to the increase of SBS of GICs containing nGO. Also, the addition of nGO to GICs can increase cohesive failure in the cement in the case of nonuniform distribution and the formation of nanoparticle mass accumulation areas, which in turn leads to weak points and porosity in the cement (Abdulkader & Aljubori, 2022; Sharafeddin, Alavi, et al., 2020).

The thermocycling process was used to mimic the temperature and humidity changes in the oral dynamic environment, which all affect the bond strength of restorative materials to the dentin. As the number of cycles increases, the strength of the bond decreases as a result of the thermal stress created in the interface and the absorption of more moisture (Ballal, 2019).

The application of GICs in restorative treatments is limited due to their poor mechanical and physical properties. Increasing the bond strength of GICs to the tooth and improving its mechanical properties lead to wider clinical use of this material in the restoration of the posterior teeth as a permanent restoration material; therefore, there is a possibility of improving the prognosis of these restorations. Promising studies have been conducted in this field. However, to provide more confident opinions about the clinical use of these restorative materials, more extensive research is needed with the use of more precise techniques (Sari & Ugurlu, 2023).

However, the simulation of different mouth conditions, including chewing forces and chemical changes caused by acidic foods, was among the limitations of this study. We followed the manufacturer's instructions for mixing the powder with the liquid, while the addition of GO might require changes in these proportions. Given the promising results of this study and the need to improve the bond strength of GICs for use in conservative restorations of the posterior teeth, it is suggested that more extensive research should be conducted with different percentages of GO and different ratios of powder to liquid and different methods of mixing GO with the cement to improve its uniform distribution as well as in vivo studies to achieve more accurate and clear results.

## 5 CONCLUSIONS

Considering the limitations of this study, the following results are extracted:

1‐ The addition of 1 wt.% and 2 wt.% nGO significantly increased the SBS of CGIC and RMGIC to the dentin.
2‐ Increasing the amount of nGO to 2 wt.% did not significantly increase the SBS of the CGIC/RMGIC + 1 wt.% nGO.
3‐ The SBS of the RMGIC groups was significantly higher than their corresponding CGIC groups.
4‐ The difference in SBS of RMGIC with CGIC + 1% GO and CGIC + 2%GO was not significant.

## AUTHOR CONTRIBUTIONS

Prof. Farahnaz Sharafeddin presented the conception and designed this research. Dr. Parisa Ghodrati prepared the specimens and collected the data under the supervision of Prof. Farahnaz Sharafedin. Dr. Naimeh Al‐Sadat Ethmarian from the Dental Research Development Center at Shiraz Dental Schools analyzed, and Prof. Farahnaz Sharafeddin and Dr. Parisa Ghodrati interpreted the data. Prof. Farahnaz Sharafeddin and Dr. Parisa Ghodrati drafted the article. Prof. Farahnaz Sharafeddin revised the article. Dr Nasrin Shokrpour from the Research Consultation Center of Shiraz University of Medical Sciences edited the language of the article.

## CONFLICT OF INTEREST STATEMENT

The authors declare no conflict of interest.

## ETHICS STATEMENT

Vice‐Chancellor of Shiraz University of Medical Science, Shiraz, Iran, approved this research with the ethical approval code (IR.SUMS.DENTAL.REC.1401.029).

## Data Availability

The data supporting this research article were represented at the time of submission.
